# Dysbiosis and metabolic pathway shifts in the gut microbiome of children with sepsis: a comparative analysis

**DOI:** 10.3389/fmicb.2025.1715990

**Published:** 2026-01-12

**Authors:** Jiayue Xu, Jiru Li, Xiangmei Kong, Chen Zhang, Baocui Qi, Xiaodong Zhu, Yueniu Zhu, Yaya Xu

**Affiliations:** 1 Department of Pediatric Critical Care Medicine, Xinhua Hospital Affiliated with the Medical School of Shanghai Jiao Tong University, Shanghai, China; 2 Department of Medicine, Dinfectome Inc., Nanjing, China; 3 Bioinformatics and System Development Department, Dinfectome Inc., Nanjing, China

**Keywords:** gut microbiome dysbiosis, KEGG pathway, metagenomic next-generation sequence, pediatric, Sepsis

## Abstract

**Background:**

The newly published Phoenix Sepsis Score in 2024 for assessing sepsis in children mainly focuses on respiratory, cardiological, coagulation and neurological indicators, whereas the gut microbiome also plays key roles in the occurrence and progression of sepsis. Additionally, emerging evidence suggests that specific biomarkers in gut microbiome are associated with disease progression. This study aimed to explore the differences in gut microbiome diversity, composition and function between septic and healthy children, and to establish correlations with clinical indicators and outcomes, providing new possibilities for the diagnosis and treatment of sepsis.

**Results:**

Analysis of gut microbiome was performed in 20 sepsis children and 9 healthy controls aged between 3 and 18 years old. The anal swab samples were analyzed by metagenomic next-generation sequencing. Significant differences were observed in *α* and *β* diversity of gut microbiome between sepsis group and healthy controls groups. Especially, Shannon diversity was significantly correlated with white blood cell count, serum lactate, length of pediatric intensive care unit stay and length of hospital stay (all *R* > 0, *p* < 0.05). *Firmicutes* and *Bacteroidetes* were both dominant in most of children in SG and HC groups, while three in SG showed extremely low combined abundances of *Firmicutes* and *Bacteroidetes* (<10%), which might be associated with chemistry therapy and death outcome. Bacteria associated with nosocomial infections, including genus taxa *Acinetobacter*, *Prevotella*, *Escherichia*, *Klebsiella*, *Bacteroides*, and *Corynebacterium*, can be dominant (relative abundance>70%) in sepsis group, which were absent in healthy control group. *Enterococcus* abundance not only predicted sepsis risk (AUC = 0.85) but also was correlated with 28-day mortality (*R* > 0, *p* = 0.004). Gene function prediction based on Kyoto Encyclopedia of Genes and Genomes pathway analysis indicated significant differences profile in SG and sepsis-deaths groups. The enriched gut microbiome genes were related to cellular proliferation, energy metabolism, signal transduction, the oxidative stress response and arginine metabolism.

**Conclusion:**

Significant differences in diversity, taxa composition and gene function in the gut microbiome existed between septic and healthy children. The associations between gut microbiome dysbiosis and clinical indicators were identified. *Enterococcus* could be a biomarker to predict sepsis risk.

## 1 Introduction

Sepsis, characterized by a dysregulated host response to infection, is a major contributor to global childhood morbidity and mortality, with approximately 25 million pediatric cases and up to 3 million fatalities annually (Rudd et al., 2020). The recently published International Consensus Criteria for Pediatric Sepsis and Septic Shock (2024) introduces the Phoenix Sepsis Score, a new tool for assessing organ function in septic children that focuses on the respiratory, cardiovascular, coagulation, and neurological systems (Schlapbach et al., 2024). The implementation of these criteria is crucial for the identifying pediatric patients with confirmed or suspected sepsis who are at elevated risk of mortality during the initial phases of disease progression. The gut microbiome plays key roles in maintaining human health through interactions between its microbiota and host immune, metabolic, and defense processes (Fan and Pedersen, 2021; Gu et al., 2023). It is commonly observed that the dysbiosis of gut microbiome are associated with the development and progression of sepsis (Klingensmith and Coopersmith, 2023; Miller et al., 2021; Yang et al., 2024; Mankowski et al., 2022; Bongers et al., 2023; Sun et al., 2023). Evidence shows that during sepsis, the gut ecosystem may experience dysbiosis, characterized by the dominance of pathogenic genera such as *Clostridium* and *Enterococcus*, while beneficial microbiota, particularly those producing short-chain fatty acids, decrease (Adelman et al., 2020; Dickson et al., 2016). Specific changes in the gut microbiome of sepsis patients, especially colonization by *Enterococcus* species and other ICU-prevalent nosocomial pathogens, correlate with infection incidence and mortality (Agudelo-Ochoa et al., 2020; Freedberg et al., 2018). Nevertheless, the compositional complexity and variability of the gut microbiome, even within healthy populations, present significant challenges in elucidating the etiology and pathogenesis of diseases (Martinez-Guryn et al., 2019; Schlechte et al., 2023). The current understanding of these alterations remains insufficient for precise pediatric sepsis diagnosis and management guidelines. Therefore, we hypothesize that early-stage analysis of gut microbiota diversity, composition and metabolic pathways in sepsis patients may facilitate a more comprehensive clinical evaluation and improve prognostic outcomes. Based on this hypothesis, we conducted a retrospective, observational study to investigate these potential associations.

This study employed metagenomic next-generation sequencing (mNGS) to analyze the gut microbiota profiles of 20 pediatric sepsis patients and 9 healthy controls. The primary objective was to ascertain whether statistically significant differences existed in the gut microbiota between the sepsis group (SG) and healthy controls (HC) groups. Furthermore, this investigation sought to elucidate the associations between gut microbiota alterations and clinical parameters, as well as to explore the potential metabolism pathways shifts in pediatric sepsis children.

## 2 Materials and methods

### 2.1 Participant selection, fecal specimens, and clinical data collection

**Figure 1:**
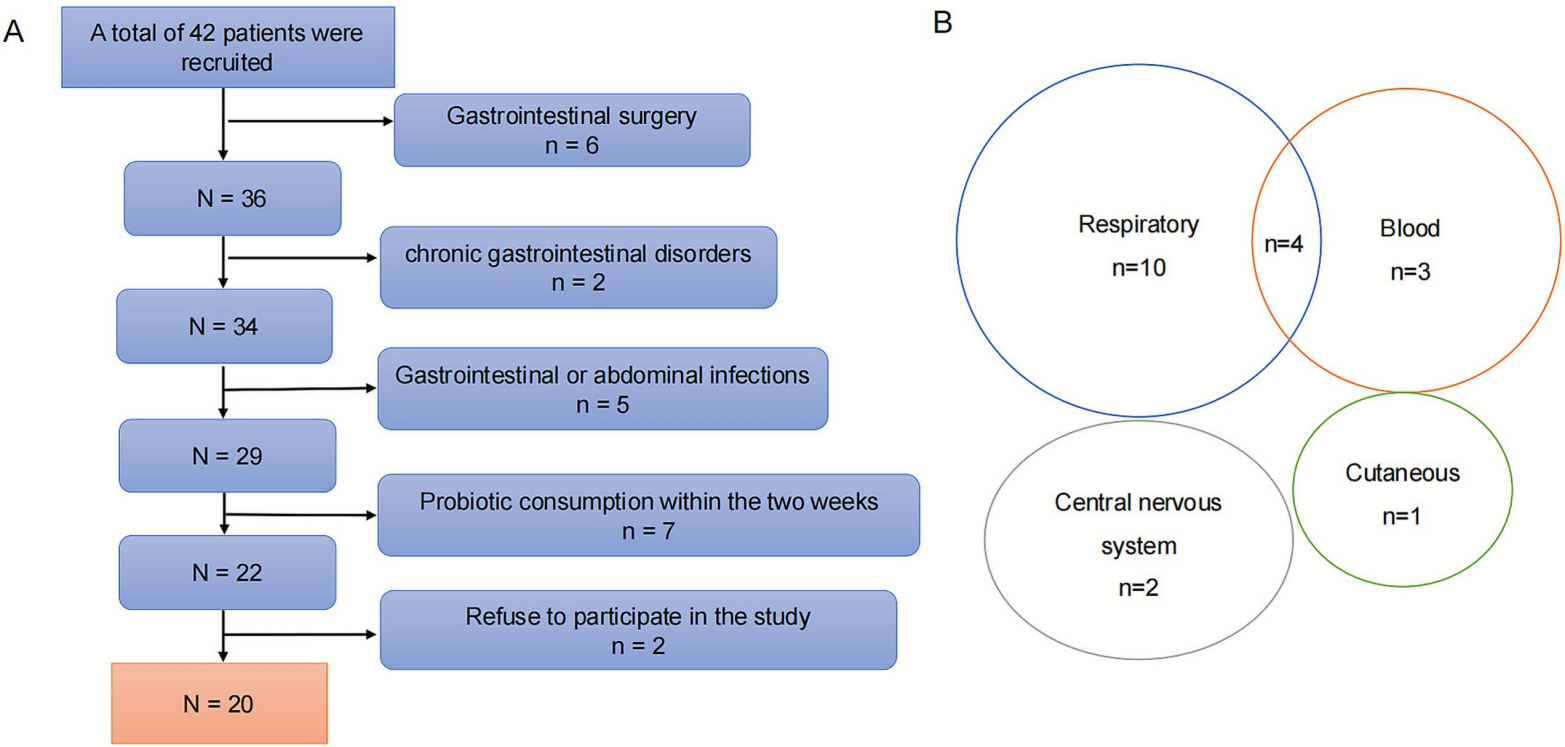
**(A)** Flow diagram of patient recruitment. **(B)** Venn diagram of infection locations in sepsis children.

This study received ethical approval from the Ethics Committee of Xinhua Hospital Affiliated with Shanghai Jiao Tong University School of Medicine (Ethics No. XHEC-D-2022-255). The investigation was designed as a retrospective observational study and was conducted in the pediatric intensive care unit (PICU) of the institution (Clinical trial number: NCT06197828). The study population comprised children aged between 3 and 18 years who fulfilled the Phoenix sepsis criteria from March 2021 to March 2022. Subjects were excluded if they (1) had gastrointestinal surgery; (2) had chronic gastrointestinal disorders; (3) presented with gastrointestinal or abdominal infections; (4) had a documented history of probiotic consumption within the preceding 2 weeks; (5) refused to participate in the study. A total of 20 pediatric patients were ultimately included in the SG (Oren and Garrity, 2021), and 9 healthy children with no documented exposure to probiotics or antibiotics during the preceding 2 months were recruited as the HC group (Figure 1A). For the SG, fecal samples were obtained within 24 h of sepsis diagnosis. The methodology for fecal sample collection and preservation has been described in our prior publication (Xu et al., 2023).

Clinical data were obtained concurrently with anal swabs. Patients were followed for 28 days after enrollment in the study. The principal outcome measures included the 28-day mortality rate and length of PICU stay (LOIS).

### 2.2 Metagenomic next-generation sequencing and statistical methods

Prior to performing mNGS, DNA was extracted, assessed, and used to construct libraries. High-quality sequencing data were obtained by filtering out low-quality reads, adapter contaminants, duplicates, and short reads (<36 bp). Microbial taxonomy was identified via Kraken (v2.0.7), and species abundance was estimated via Bracken (v2.5.0). This methodology follows our previously published research (Xu et al., 2023), which provides a more detailed description of the approach.

Statistical analyses were performed via R (v3.6.0) for diversity indices. Alpha diversity (Shannon and Chao1) was used to assess community richness and evenness. Beta diversity was assessed via Bray–Curtis dissimilarity matrices. Permutational multivariate analysis of variance (PERMANOVA) was used to test for group differences, whereas principal coordinate analysis (PCoA) was used to visualize *β* diversity. Differential abundance analysis was performed via the Kruskal–Wallis test (implemented in the R package ‘kruskal.test’). Linear discriminant analysis effect size (LEfSe) was employed to identify potential biomarkers in the sepsis group. Features with an absolute linear discriminant analysis (LDA) score exceeding 2.0 were considered statistically significant. Septic children were stratified into high-diversity (Shannon index ≥2.06) and low-diversity (Shannon index <2.06) groups on the basis of the mean Shannon index observed in healthy control subjects.

Statistical analyses of the clinical data were performed via SPSS software (v25.0). Continuous variables are presented as the means ± standard deviations for normally distributed data or medians (interquartile ranges) for nonnormally distributed data. Spearman’s rank correlation coefficient was used to examine associations between clinical variables and gut microbiota characteristics. *p* values <0.05 were considered statistically significant for all tests.

## 3 Results

### 3.1 Demographic and clinical characteristics of the study cohort

The demographic characteristics of the SG and HC are shown in Table 1. In the SG, 45% of the children presented with comorbidities, including leukemia, solid neoplasms, genetic metabolic diseases, and hemophagocytic lymphohistiocytosis. Upon enrollment, 90% of the patients were receiving broad-spectrum antimicrobial therapy. Additionally, 80% of the patients maintained enteral nutrition, and 35% required mechanical ventilatory support. In terms of infection sites, the respiratory system emerged as the predominant focus of infection, followed by the bloodstream, the central nervous system, and cutaneous infections. Notably, 4 patients presented with multiple infection sites (Figure 1B). Respiratory pathogens were isolated from 7 patients, with the following distributions: *Acinetobacter baumannii* (*n* = 3), *Klebsiella pneumoniae* (*n* = 1), *Streptococcus pneumoniae* (*n* = 1), and *Moraxella catarrhalis* (*n* = 1). One patient had polymicrobial respiratory infection involving *Acinetobacter baumannii*, *Pseudomonas aeruginosa*, *Staphylococcus aureus*, and *Candida tropicalis*. Additionally, blood cultures yielded positive results in two patients: one for *Candida parapsilosis* and another for *Staphylococcus hominis* (Supplementary Table S1). The median duration of LOIS for the sepsis group was 7.5 days, with a median LOS period of 19.5 days. During the follow-up period, 4 patients died due to multiple organ dysfunction syndrome (MODS). The HC group comprised 5 females and 4 males, with a mean age of 8.78 ± 0.87 years.

**Table 1:** Characteristic information of sepsis and healthy control groups.

Characteristic	SG (*n* = 20)	HC (*n* = 9)
Basic information		
Age, year	6.74 ± 3.36	8.78 ± 0.87
Sex [*n*, Male/Female]	10/10	4/5
BMI (kg/m^2^)	16.12 ± 2.71	17.21 ± 1.32
Basic disease [*n*, (%)]		
Solid tumor	4 (20.0)	/
Leukemia	3 (15.0)	/
Hemophagocytic syndrome	1 (5.0)	/
Congenital genetic metabolic disease	1 (5.0)	/
None	11 (55.0)	/
Treatments [*n*, (%)]		
Broad-spectrum antibiotics	18 (90.0)	
Enteral nutrition	16 (80.0)	
Mechanical ventilation	7 (35.0)	
Infection site [*n*, (%)]		
- Respiratory	14 (70.0)	
- Blood	7 (35.0)	
- Central nervous system	2 (10.0)	
- Cutaneous	1 (5.0)	
- Multi-site infection	4 (20.0)	/
Prognosis		
28-day mortality rate	4 (20%)	/
Incidence of infection confirmed by bacterial culture	10 (50%)	/
LOIS (days)	7.50 (4.00, 14.75)	/
LOS (days)	19.50 (12.00, 31.75)	/

BMI, body mass index; LOIS, Length of PICU stay; LOS, Length of hospital stay.

### 3.2 Substantial disparities in the gut microbiome diversity between pediatric sepsis patients and healthy controls

**Figure 2:**
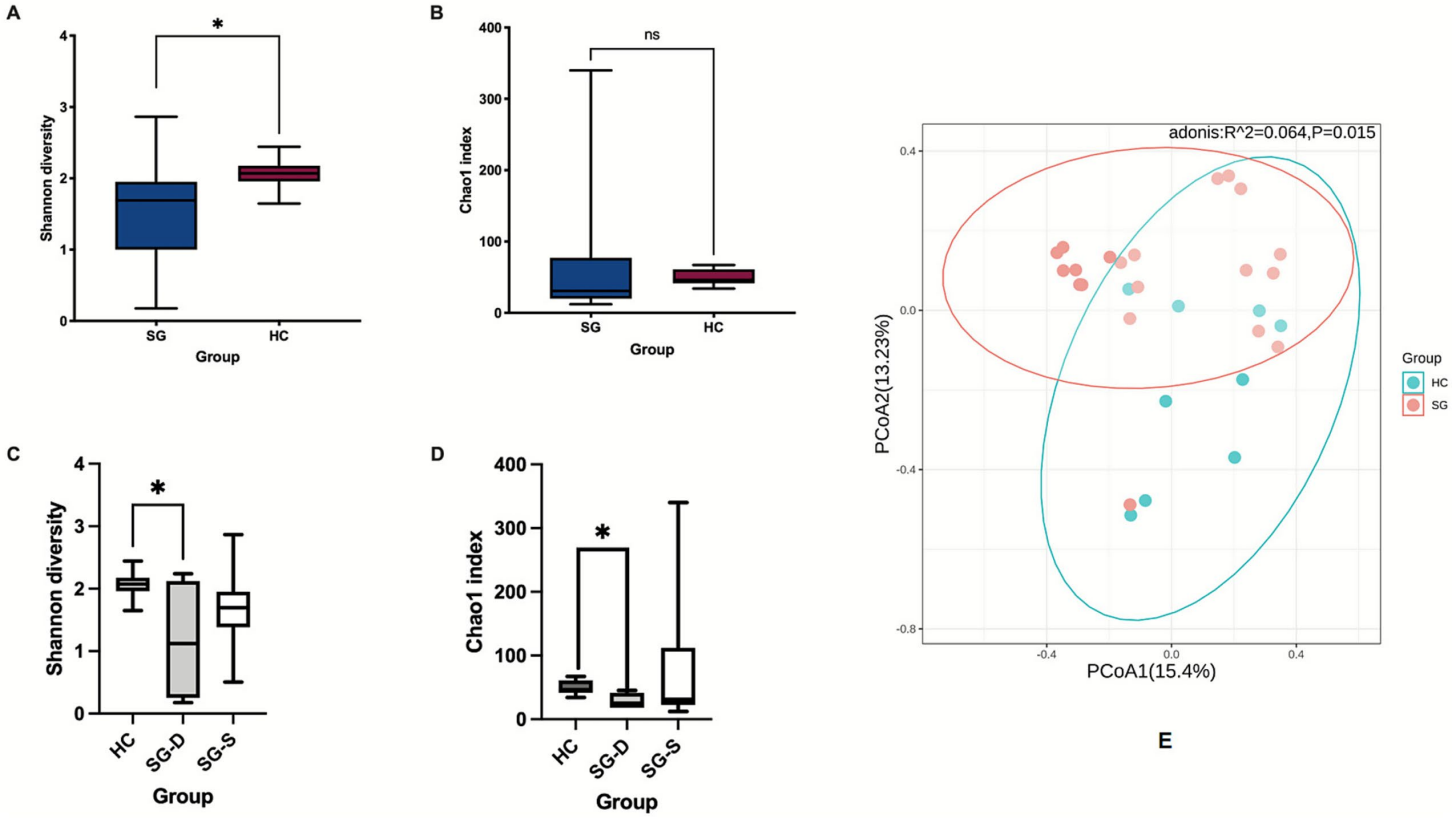
The *α* and *β* diversity analysis of the microbiota in the sepsis and healthy control groups. **(A,B)** Shannon and Chao1 indexes analysis between SG and HC groups. **(C,D)** Shannon and Chao1 indexes analysis among HC, SG-D, and SG-S groups. **(E)** PCoA of beta diversity based on the unweighted UniFrac distance between SG and HC groups. SG, sepsis group; HC, healthy control; SG-D, sepsis-deaths; SG-S, sepsis *s*urvival*s*. *p < 0.05.

The Shannon index was significantly lower in the SG compared to the HC (1.51 ± 0.67 vs. 2.06 ± 0.23, *p* < 0.05) (Figure 2A), while the Chao1 index showed a decreasing trend in the SG (Figure 2B). Among sepsis patients, sepsis-survivors (SG-S) exhibited slightly greater alpha diversities than SG-D, although the differences were not statistically significant (Figures 2C,D). In contrast, both the Shannon and Chao1 indices were significantly lower in SG-D than in HC (Shannon: 1.16 ± 0.99 vs. 2.06 ± 0.21; Chao1: 28.25 ± 12.63 vs. 49.44 ± 11.28; both *p* < 0.05) (Figures 2C,D). PCoA visualization supported by PERMANOVA analysis showed that *β* diversity of the gut microbiome communities differed significantly between the SG and HC groups (Figure 2E *p* = 0.015).

### 3.3 Gut microbiome compositional shifts between sepsis patients and healthy controls

**Figure 3:**
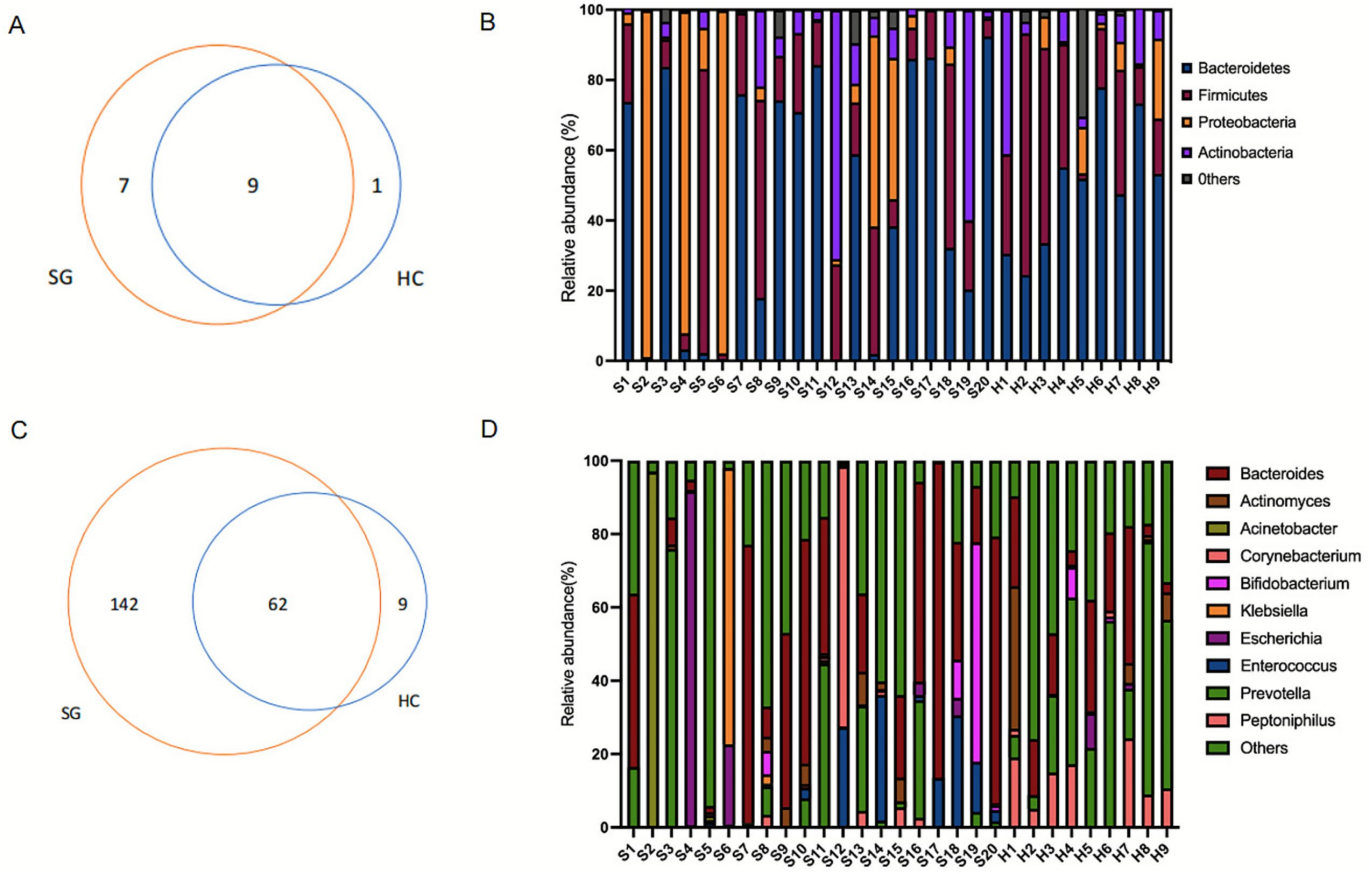
**(A,B)** Taxa venn diagram and relative abundance of different taxa at the phylum level between sepsis and healthy control groups. **(C,D)** Genera Venn and relative abundance of different genera at the genus level between sepsis and healthy control groups.

Phylum-level analysis of the microbial composition revealed 9 shared taxa between the SG and HC groups. The SG contained 7 unique taxa, while the HC contained only 1 unique taxon (Figure 3A). Both groups exhibited high relative abundances of *Firmicutes (synonym Bacillota)* and *Bacteroidetes (synonym Bacteroidota)* (Aharon and George) (Figure 3B). In the HC group, the cumulative relative abundance of these two phyla ranged from 53.46 to 94.86% (Figure 3B). Although the median cumulative abundance did not differ significantly between the groups [SG vs. HC: 83.96% (38.78%, 95.89) vs. 83.96% (63.99%, 91.84), *p* = 0.96], three patients in the SG exhibited remarkably low combined abundances of *Firmicutes* and *Bacteroidetes* (<10%) (Figure 3B). Notably, two of these patients did not survive during the follow-up period. Patient S2, diagnosed with mucopolysaccharidosis, developed infection following chemotherapy and hematopoietic stem cell transplantation. Patient S6, diagnosed with hemophagocytic syndrome, contracted infection after chemotherapy. The third patient, S4, presented with brain herniation and respiratory failure.

At the genus level, the SG displayed more unique taxa compared to the HC (Figure 3C). In contrast to no single genus exceeded a relative abundance of 70% in any control subject, eight patients in the SG had gut microbiomes dominated by a single genus (*Acinetobacter* spp., *Prevotella* spp., *Escherichia* spp., *Klebsiella* spp., *Bacteroides* spp., and *Corynebacterium* spp.), with relative abundances ranging from 70.95 to 96.96% (Figure 3D). Notably, patient S2, who had *Acinetobacter baumannii* isolated from bronchoalveolar lavage fluid, also showed gut colonization by *Acinetobacter* spp. (relative abundance of 96.96%, Supplementary Table S1). Furthermore, compared to the HC group, sepsis patients demonstrated significant alterations in bacterial composition at both genus and species levels. The abundances of *Peptoniphilus* spp., *Prevotella* spp., and *Prevotella disiens* were significantly decreased in sepsis patients. Conversely, the abundances of nosocomial pathogens, including *Enterococcus* spp., *Acinetobacter baumannii*, *Enterococcus faecalis*, and *Bacteroides caccae*, were significantly higher than in controls (Supplementary Figure 1S-A).

LEfSe analysis identified significant differences in the bacterial species distributions between the SG and HC groups across multiple taxonomic levels (Supplementary Figure 1S-B). These findings suggest that the gut microbiome composition patterns may potentially serve as a novel adjunctive diagnostic tool for sepsis.

### 3.4 Association between gut microbiome alterations and clinical parameters in pediatric sepsis patients

**Figure 4:**
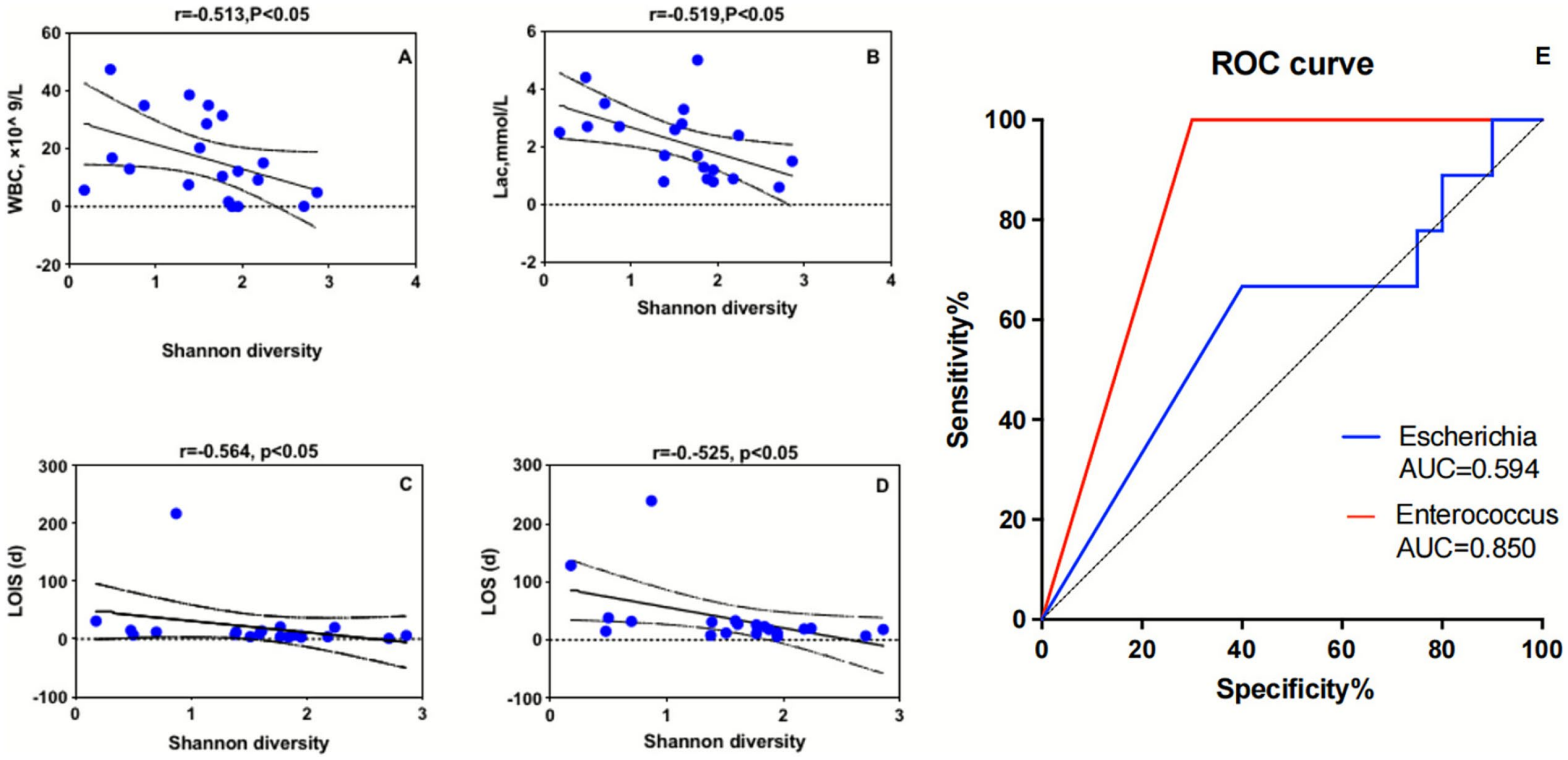
**(A–D)** Correlation analysis of Shannon diversity and clinical indicators. **(E)** ROC curve of *Enterococcus* and *Escherichia* for distinguishing sepsis. ROC, Receiver operating characteristic; AUC, area under the curve.

The SG was stratified into high-diversity (Shannon index ≥2.06) and low-diversity (Shannon index <2.06) groups. Demographic characteristics, including age, sex, and body mass index (BMI), showed no significant differences between these groups (Table 2). However, patients in the low-diversity group presented slightly elevated with white blood cell count (WBC), serum lactate (Lac), and longer LOIS and length of hospital stay (LOS) compared to the high-diversity group (Table 2). Further correlation analyses revealed significant inverse relationships between Shannon diversity and serum Lac concentrations, WBC, LOIS, and LOS (*p* < 0.05, Figures 4A–D). Additionally, there was a significant association between the relative abundance of *Enterococcus* spp. and 28-day mortality (*p* = 0.004, Supplementary Table S2).

**Table 2:** Comparison of clinical characteristics between high and low Shannon diversity groups in sepsis patients.

Clinical characteristic	High diversity group (*n* = 12)	Low diversity group (*n* = 8)	*t*/*Z*/*χ*^2^	*p*-value
Age, year	7.33 ± 3.65	5.86 ± 2.86	0.95	0.35
Sex [Male, *n* (%)]	6 (50%)	4 (50%)	/	1.00
BMI (kg/m^2^)	15.61 ± 2.49	16.88 ± 3.01	−1.02	0.32
PCIS	90.17 ± 6.74	82.50 ± 13.48	1.69	0.11
CRP (mg/L)	127.83 ± 69.2	99.75 ± 77.06	0.85	0.41
PCT (ng/mL)	5.89 (0.50, 20.47)	1.95 (1.23, 5.98)	−0.23	0.82
WBC (×10^ 9/L)	9.86 (0.92, 21.85)	18.57 (10.28, 36.80)	−1.77	0.08
Lac (mmol/L)	1.40 (0.90, 2.60)	2.65 (2.10, 3.10)	−1.47	0.14
LOIS, day	6.00 (3.50, 11.50)	12.00 (7.50,23.00)	−1.86	0.06
LOS, day	18.5 (11.5, 24.5)	31.5 (13.5, 83)	−1.51	0.13
28 days mortality rate [*n* (%)]	2 (16.67%)	2 (25%)	/	1.00

BMI, body mass index; PCIS, pediatric critical illness score; CRP, C—reactive protein WBC, white blood cell; lac, lactic acid; LOIS, length of PICU stay; LOS, length of hospital stay. The detection of WBC was carried out through whole—blood specimens, the other biomarkers were detected from serum samples. Reference range and cut-off values: PCIS, 0–100; CRP < 8 mg/L, PCT < 0.5 ng/mL, WBC 4.3–11.3*10^9/L, lac 0.7–2.1mmo/L.

### 3.5 ROC analysis reveals *Enterococcus* as a promising biomarker for sepsis prognosis

Receiver operating characteristic (ROC) curve analysis was performed to assess the diagnostic potential of the gut microbiota taxa for sepsis (Figure 4E). The results revealed that *Enterococcus* spp. exhibited a significant area under the ROC curve (AUC) of 0.850 [95% confidence interval (CI): 0.7122–0.9878, *p* = 0.003], indicating strong discriminatory power between sepsis patients and healthy controls. In contrast, *Escherichia* spp. demonstrated a lower AUC of 0.594 [95% CI: 0.3856–0.8303, *p* = 0.4229], suggesting limited diagnostic value in our cohort. These findings suggest that elevated relative abundance of *Enterococcus* spp. may be associated with sepsis, warranting further investigation as a potential diagnostic indicator.

### 3.6 Altered microbial gene abundances in KEGG pathways associated with sepsis status and survival outcomes

**Figure 5:**
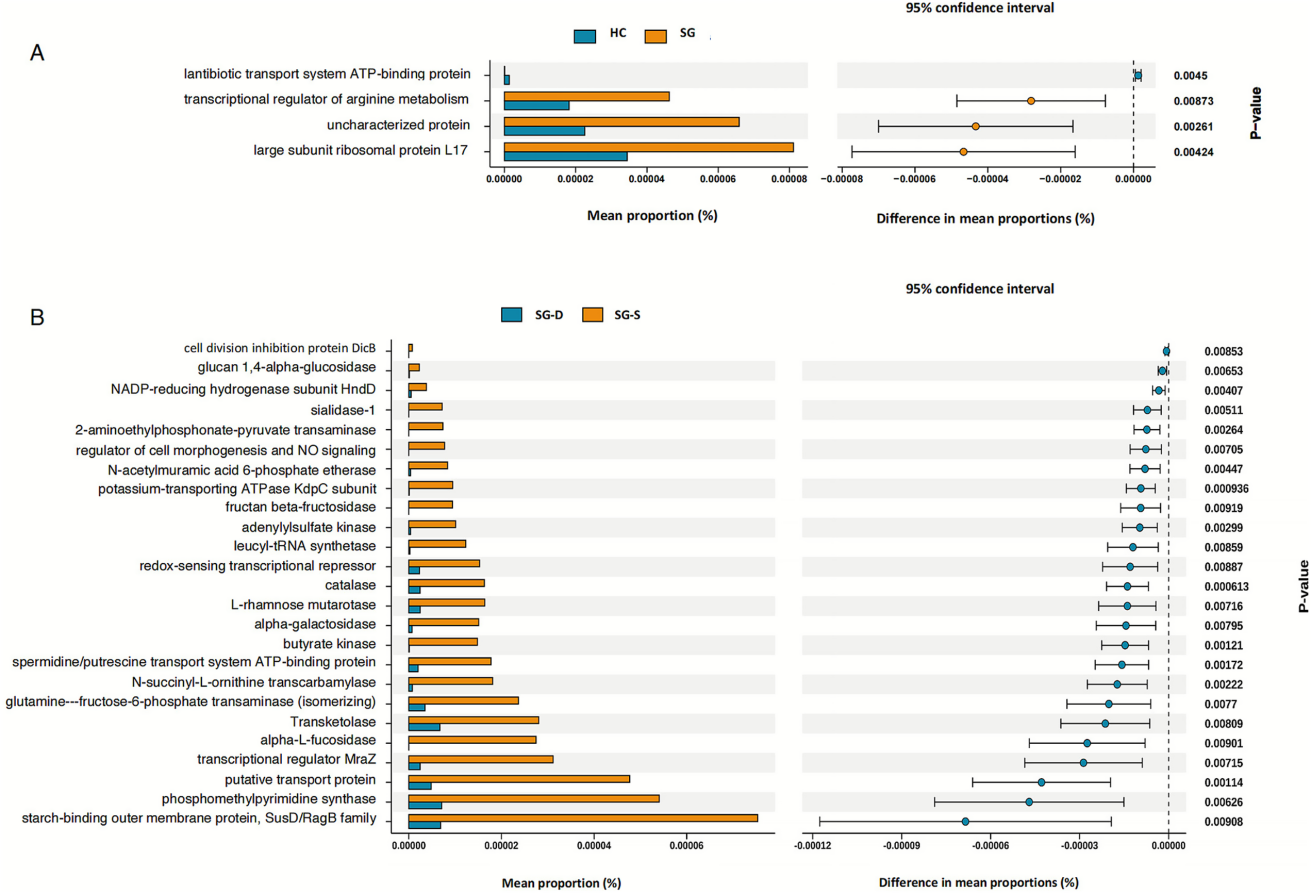
KEGG pathways analysis based on gut microbiome gene function prediction. **(A)** KEGG pathways analysis between the SG and HC. **(B)** KEGG pathways analysis between SG-D and SG-S. The histograms on the left represent the names of KEGG pathways and their relative abundances, and the dot plots on the right represent the corrected *p*-values. Corrected *p* < 0.05 was considered significant and retained. SG, epsis group; HC, healthy controls; SG-D, sepsis-deaths; SG-S, sepsis-survivals; KEGG, Kyoto encyclopedia of genes and genomes.

We performed functional analysis comparing KEGG pathway associated-gene abundances between SG and HC groups. Our findings revealed a modest enrichment of genes encoding the lantibiotic transport system ATP-binding protein in healthy children. In contrast, children with sepsis exhibited significant enrichment of genes encoding the transcriptional regulator of arginine metabolism, an uncharacterized protein, and the large subunit ribosomal protein L17 (Figure 5A). Further stratification analysis comparing sepsis survivors and non-survivors revealed significant downregulation of genes encoding regulators of cell morphogenesis, nitric oxide signaling pathways, carbohydrate-metabolizing enzymes (including glucan 1,4-alpha-glucosidase, fructan beta-fructosidase, alpha-L-fucosidase, and alpha-galactosidase), and redox-sensing transcriptional repressors in non-survivors (Figure 5B). These functional alterations in the gut microbiome may potentially compromise host physiological homeostasis, particularly in patients with poor clinical outcomes.

## 4 Discussion

Our investigation revealed significant differences in the gut microbiota composition and structure between the SG and HC groups. These differences include reduced microbial diversity and evenness, decreased abundance of beneficial bacteria, and increased pathogenic microorganisms, consistent with previous findings in adult sepsis patients and critically ill individuals (Agudelo-Ochoa et al., 2020). *Firmicutes* and *Bacteroidetes* typically constitute more than 90% of the healthy gut microbiome (Jandhyala et al., 2015). While we did not detect significant differences in the overall abundance of these phyla between SG and HC, we observed greater inter-individual variability in these dominant phyla among pediatric sepsis patients. Notably, three sepsis patients exhibited dramatically reduced proportions of combined *Firmicutes* and *Bacteroidetes* (F + B) (<10%), with two of these patients having developed secondary infections following chemotherapy, and two dying during follow-up. These observations align with findings by Ojima et al. (2016) who demonstrated that *Firmicutes* and *Bacteroidetes* abundances in ICU patients vary considerably between individuals and fluctuate dynamically over time. Furthermore, evidence indicates that a low *Firmicutes/Bacteroidetes* (F/B) ratio may serve as a potential biomarker to distinguish between children with sepsis/septic shock and healthy controls (Sankar et al., 2024). The association between altered relative abundance of *Firmicutes* and *Bacteroidetes* and poor clinical outcomes suggests that gut microbiome composition warrants further investigation as a potential indicator of disease severity and prognosis in pediatric sepsis, although larger prospective studies are needed to validate these preliminary observations.

Despite no difference in the Chao1 index between the SG and HC cohorts, genus-level analysis revealed heterogeneity in the gut microbiota distribution within the SG. Notably, 40% of the pediatric sepsis patients exhibited gut microbiota dominated by a single genus, including nosocomial pathogens such as *Acinetobacter* spp., *Escherichia* spp., and *Klebsiella* spp. This finding is particularly relevant in subject S2, where *Acinetobacter baumannii* was identified in both bronchoalveolar lavage fluid and as the predominant microorganism in the gastrointestinal tract, suggesting potential gut-lung microbial translocation. This phenomenon has been demonstrated in preclinical studies using cecal ligation and puncture (CLP)-induced sepsis murine models, which have shown rapid accumulation of gut-associated microbiota in the lower respiratory tract (Dickson et al., 2016). Consistent with our observations, Dickson et al. (2020) reported that 41% of acute respiratory distress syndrome patients exhibited pulmonary colonization by gut-predominant microbes, a phenomenon absent in healthy controls. This gut-lung microbial translocation was characterized by the presence of the most abundant gut microbial genera in the pulmonary microbiome. Furthermore, Dickson et al. (2016) demonstrated that lung microbiota dysbiosis was associated with increased tumor necrosis factor-*α* (TNF-α) production, which promotes inflammatory responses in lung tissue and may contribute to lung injury. Collectively, these findings suggest that gut dysbiosis in pediatric sepsis patients, particularly single-genus dominance, may have clinical implications beyond the gastrointestinal tract, potentially contributing to respiratory complications through gut-lung microbial translocation (Dickson et al., 2020).

Our analysis revealed increased gut microbiota taxa in sepsis patients compared with healthy controls, both at the phylum and genus levels. The altered gut microbiome in sepsis, influenced by both disease pathophysiology and therapeutic interventions, may promote the growth of typically subdominant bacterial species. Our study demonstrated increased relative abundance of *Prevotella disiens* and *Bacteroides caccae*, opportunistic pathogens that potentially exert adverse effects on gastrointestinal health through induction of intestinal inflammation and alteration of mucosal barrier integrity (Larsen, 2017; Lavoie et al., 2019). These organisms can emerge as opportunistic nosocomial pathogens, often exhibiting multidrug resistance phenotypes, thereby posing significant challenges in the management of hospital-acquired infections (Rogers et al., 2016).

Our study revealed a significant increase in *Enterococcus species* in the SG, which was positively correlated with 28-day mortality. Rogers et al. (2016) also reported a significantly greater prevalence of *Enterococcus* in ICU patients than in healthy controls. Similarly, Sun et al. (2023) reported a significant increase in the relative abundance of *Enterococcus species* among sepsis patients and subsequently corroborated this observation through rigorous experimentation in animal models. Our findings align with those of Freedberg et al. (2018) who reported that *vancomycin-resistant Enterococcus* (VRE) colonization and *Enterococcus* dominance (relative abundance ≥30%) were significantly associated with mortality and infection events. Moreover, our LEfSe analysis identified *Enterococcus* spp. as a potential biomarker for sepsis diagnosis (Supplementary Figure 1S-B), which was supported by the ROC curve analysis (Figure 3E). While previous studies have linked *Escherichia coli* to increased mortality in sepsis patients (Maldonado et al., 2024) and highlighted *Escherichia/Shigella* as a diagnostic indicator in neonatal late-onset sepsis (Ma et al., 2024). While our cohort did not show high diagnostic efficacy for *Enterobacteriaceae* spp. in sepsis.

Importantly, antibiotic exposure is a key potential confounder in microbiome analyses of critically ill patients. Our previous work has shown that even short-term use of broad-spectrum antibiotics in critically ill children can alter gut microbial community structure and function and induce the expansion of antibiotic resistance genes (Xu et al., 2023). Thus, antibiotic exposure is also a major contributor to the observed shifts in the gut microbiome of children with sepsis. To mitigate this influence in future studies, careful control of sampling windows (e.g., collecting specimens prior to or within narrowly defined intervals after antibiotic initiation) and stratification by antibiotic use (class, spectrum, timing, dose, duration, and combination therapy) will be essential. These measures will facilitate a clearer understanding of the characteristic features of the gut microbiome in pediatric sepsis.

In our comparative analysis of KEGG pathways associated with the gut microbiota, we found significant differences between the SG and HC groups. The SG resulted in the upregulation of ribosomal components involved in protein biosynthesis and transcriptional regulators of arginine metabolism. L-arginine, a precursor for nitric oxide (NO) synthesis, plays a crucial role in various physiological processes (Wu et al., 2021). Conversely, the sepsis mortality cohort exhibited downregulation of cell morphogenesis regulators and NO signaling mediators. During early sepsis, adequate NO derived from endothelial NOS supports microvascular perfusion and barrier integrity, whereas dysregulated inducible NOS activity can promote vasoplegia and mitochondrial dysfunction (Plummer and Bellomo, 2022). Thus, microbiome-driven shifts in arginine metabolism may influence the balance between protective endothelial NO signaling and deleterious iNOS-mediated NO excess, potentially affecting hemodynamics and tissue oxygenation (Stayer et al., 2025). By contrast, in the mortality subgroup we observed downregulation of pathways related to cell morphogenesis and mediators of NO signaling, together with broader suppression of modules involved in cellular proliferation/regulation, core metabolic processes, energy metabolism, oxidative stress responses, and signal transduction. (Wang et al., 2023). The metabolic signals align with this trajectory. Suppression of enzymes central to carbohydrate metabolism and energy homeostasis is consistent with the metabolic shutdown described in sepsis-induced immunoparalysis and organ failure. Notably, reduced butyrate kinase implies diminished microbial butyrate synthesis. Lower butyrate production may impair barrier function, facilitate bacterial translocation, and skew systemic immunity toward a maladaptive inflammatory/immune-suppressive state (Salvi and Cowles, 2021). Together with attenuated redox defenses, this pattern suggests a shift from a metabolically supportive, barrier-protective microbiome toward a community that is less capable of sustaining epithelial integrity and host anti-oxidative and vasoregulatory tone. Additionally, the mortality group presented a significant reduction in redox-sensing transcriptional repressors and catalase expression, suggesting dysregulation of redox homeostasis and impaired elimination of intracellular hydrogen peroxide. These alterations may exacerbate cellular oxidative stress, potentially accelerating apoptosis and necrosis in sepsis patients (Sahoo et al., 2024).

It should be noted that the gut ecosystem comprises archaea, fungi, viruses (including bacteriophages), and their metabolites beyond bacteria (Ouyang et al., 2024), all of which may contribute to the outcomes observed in this study. Bacteriophages can remodel bacterial communities and facilitate antimicrobial resistance gene transfer through lysogenic–lytic cycling (Cao et al., 2022). Fungi (e.g., Candida spp.) engage in cross-kingdom interactions with *Enterobacteriaceae* and *Enterococcus*, amplifying mucosal inflammation and microbial translocation (Schamberger and Plaetzer, 2021; Lv et al., 2021). Methanogenic archaea participate in hydrogen turnover, potentially altering short-chain fatty acid production and host energy homeostasis (Vemuri et al., 2020). Microbial metabolites such as butyrate, indole derivatives, trimethylamine N-oxide (TMAO), and polyamines regulate epithelial barrier integrity, innate and adaptive immune responses, and coagulation pathways, thereby plausibly influencing lactate levels, ICU length of stay, and other clinical indicators (Martinelli et al., 2024; Li et al., 2021). Given that our mNGS workflow primarily profiled bacteria, we were unable to comprehensively quantify these nonbacterial components or their metabolic outputs. Future investigations should incorporate mycobiome and virome profiling, archaeal-specific targets, and untargeted/targeted metabolomics, with multi-omics integration to delineate the coordinated roles and mechanisms of these commensals and metabolites in pediatric sepsis. Such approaches may refine risk stratification, identify trans-kingdom biomarkers, and reveal adjunct therapeutic avenues (e.g., phage-informed stewardship, antifungal modulation, and metabolite-focused interventions).

This study had several limitations: (1) Our analyses were conducted in a relatively small, single-center cohort, which limits statistical power and generalizability. As such, these results should be interpreted as exploratory and warrant confirmation in larger, prospectively enrolled multicenter studies. (2) The follow-up period was insufficient, although early gut microbiota testing at admission still provides a valuable clinical reference due to high mNGS costs. (3) The effects of pre-enrollment treatments (e.g., antibiotics) on the gut microbiomes of septic children cannot be excluded. (4) While changes in KEGG pathway expression were identified in the gut microbiota of septic children, the functional impacts of these changes were not experimentally validated. These limitations will be addressed in our future research through larger cohorts, extended follow-up periods, more detailed treatment history documentation, and functional validation studies.

## 5 Conclusion

In conclusion, significant differences in gut microbiome diversity, composition and enriched gene function were detected between septic children and healthy controls. Shannon diversity was negatively correlated with the serum lactate concentration, white blood cell count, ICU length of stay, and total length of hospital stay. The increased relative abundance of Enterococcus predicted a high risk of sepsis occurrence, with an AUC of 0.85. The expression of genes associated with KEGG pathways related to arginine metabolism, cell morphogenesis regulators and nitric oxide changed significantly between sepsis patients and non-survivors.

## Data Availability

The data presented in this study are publicly available. The data can be found at: https://www.ncbi.nlm.nih.gov, accession PRJNA1033539.

## Glossary

SG: Sepsis group
HC: Healthy controls
WBC: With white blood cell count
mNGS: Metagenomic next-generation sequencing
Lac: Lactate
LOIS: Length of pediatric intensive care unit stay
LOS: Length of hospital stay
KEGG: Kyoto Encyclopedia of Genes and Genomes
SG-D: Sepsis-deaths
SG-S: Sepsis-survivors
PICU: Pediatric intensive care unit
PERMANOVA: Permutational multivariate analysis of variance
PCoA: Principal coordinate analysis
LEfSe: Linear discriminant analysis effect size
LDA: Linear discriminant analysis
MODS: Multiple organ dysfunction syndrome;
BMI: Body mass index
PCIS: Pediatric critical illness score
PCT: Procalcitonin
ROC: Receiver operating characteristic
AUC: Area under the roc curve
CI: Confidence interval
CLP: Cecal ligation and puncture
TNF-*α*: increased tumor necrosis factor-α
VRE: *Vancomycin*-resistant Enterococcus
NO: Nitric oxide
