# Supplementary material for: Dysbiosis and metabolic pathway shifts in the gut microbiome of children with sepsis: a comparative analysis
Source: Front Microbiol. 2026 Jan 12;16:1715990. doi: 10.3389/fmicb.2025.1715990 (PMC12834510; doi:10.3389/fmicb.2025.1715990)
Supplement: Supplementary file 1 [file Data_Sheet_1.PDF]

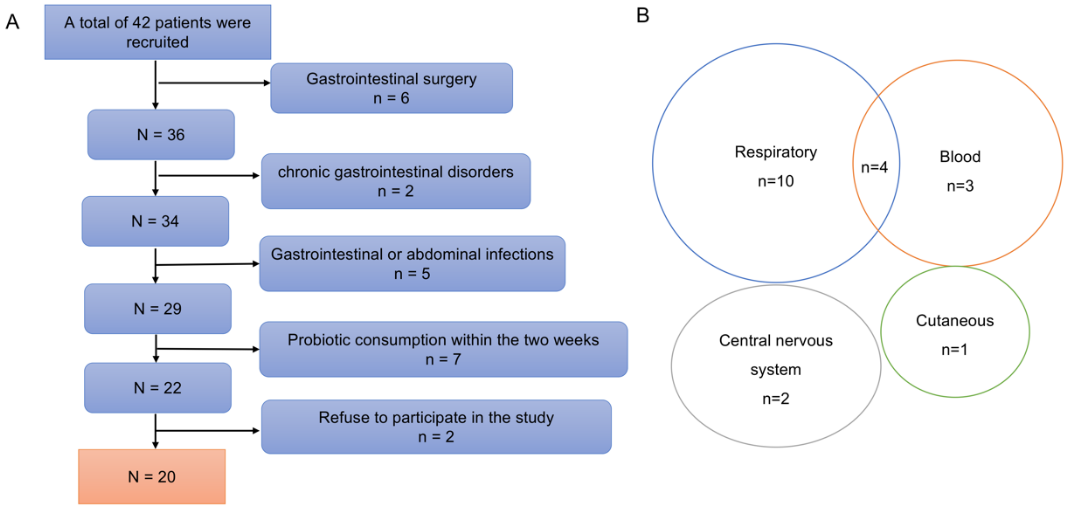

Figure 1S: A: Flow Diagram of Patient Recruitment. B: Venn Diagram of Infection locations in sepsis children.

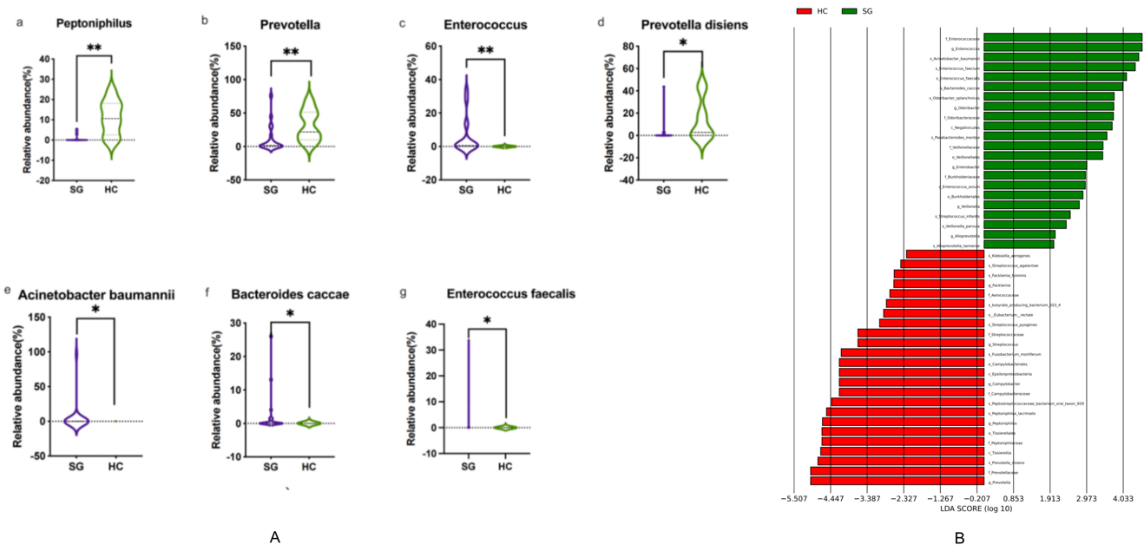

Figure 2S. A: Different bacteria at the genus and species levels in the SG and HC groups. B: Linear discriminant analysis Effect Size analysis of the SG and HC groups at the species level. The larger the absolute value of the LDA score (log 10), the greater the effect size of that bacterial species in distinguishing between the HC and SG groups. HC: healthy controls; SG: sepsis group; *P<0.05; **P<0.001.
